# Immunohistochemical Analysis of P63 Expression in Odontogenic Lesions

**DOI:** 10.1155/2013/624176

**Published:** 2013-11-24

**Authors:** Saede Atarbashi Moghadam, Fazele Atarbashi Moghadam, Sepideh Mokhtari, Ebrahim Eini

**Affiliations:** ^1^Department of Oral and Maxillofacial Pathology, Dental School of Shahid Beheshti University of Medical Sciences, Tehran, Iran; ^2^Department of Periodontology, Dental School of Sadoughi University of Medical Sciences, Yazd, Iran; ^3^Research Committee, Jundishapur University of Medical Sciences, Ahvaz, Iran

## Abstract

P63 may have a role in tumorigenesis and cytodifferentiation of odontogenic lesions. We investigated the immunohistochemical expression of P63 in a total of 30 cases of odontogenic cysts and tumors. The percentage of positive cells was calculated in the lining of odontogenic cysts and islands of ameloblastoma. P63 expression was evident in all types of odontogenic lesions. P63 was expressed throughout the lining epithelium of odontogenic keratocyst except surface parakeratinized layer. In addition, calcifying odontogenic cyst showed P63 expression in all layers. In almost all radicular and dentigerous cysts, the basal and parabasal layers were immunoreactive. Peripheral cells of ameloblastoma expressed P63; however, stellate reticulum had weaker immunostaining. No significant difference in P63 expression was observed between studied lesions (*P* = 0.86). Expression of P63 in odontogenic lesions suggests that this protein is important in differentiation and proliferation of odontogenic epithelial cells. However, it seems that it could not be a useful marker to differentiate between aggressive and nonaggressive lesions. P63 also represents a progenitor or basal cell marker, and it is not expressed in mature differentiated cells.

## 1. Introduction 

P63 is a member of P53 gene family, which has a role in epithelial development, stem cell biology, and carcinogenesis [1]. P63 is also expressed in odontogenic epithelium [2, 3]. To date, only few papers have studied the expression of this protein in odontogenic lesions [4]. It seems that the epithelial cells of aggressive odontogenic lesions have some intrinsic growth potential not present in other odontogenic lesions [5]. Therefore, understanding the pathogenesis and biological aspects of these lesions would improve the success in diagnosis and treatment procedures [6]. In this paper, we investigated the expression of P63 in various odontogenic lesions.

## 2. Materials and Methods

We studied the expression of P63 in 30 odontogenic lesions: 9 odontogenic keratocysts (OKC), 6 ameloblastoma, 6 radicular cysts (RC), 6 dentigerous cysts (DC), and 3 calcifying odontogenic cysts (COC). All were primary lesions. Histopathological diagnosis was confirmed by an experienced pathologist using H&E stained sections. Clinical data were recorded. The expression of P63 was determined by immunohistochemical staining (streptavidin-biotin peroxidase method) on paraffin sections using microwave antigen retrieval method. P63 monoclonal antibody, clone 4A4, Code N 1604, 1 : 25 dilution, Dako Cytomation, Denmark, was used. We used clone 4A4 that recognizes the ΔNP63 isoforms. The sections were incubated with primary antibody at 4°C overnight. Squamous cell carcinoma was used as positive control. For negative control, the primary antibody was replaced by a nonimmune serum. Some representative fields were randomly selected in each stained section using Olympus CX21 light microscope. Ten fields were chosen for each section. Only nuclear staining of epithelial cells was considered positive. The percentage of positive cells was calculated (In HPF) from a minimum of 1000 epithelial cells in basal-parabasal and upper layers of cysts and islands of ameloblastoma.

 SPSS software (version 16) was used, and the results were analyzed with Kruskal-Wallis and Mann-Whitney tests. Statistical significance was at *P* < 0.05.

## 3. Results

**Figure 1:**
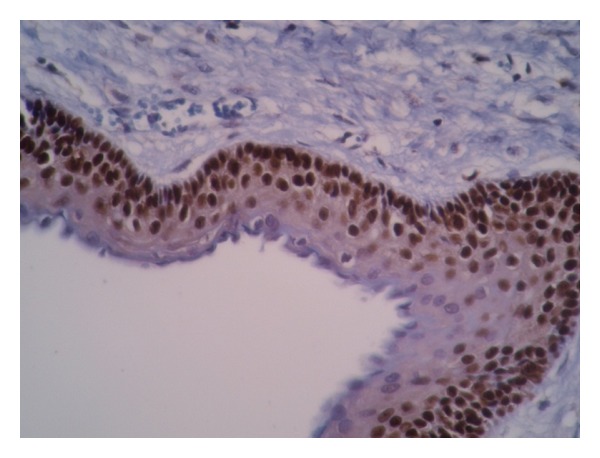
P63 is highly expressed as brown nuclei in OKC throughout the epithelial lining except parakeratinized layer (×200).

**Figure 2:**
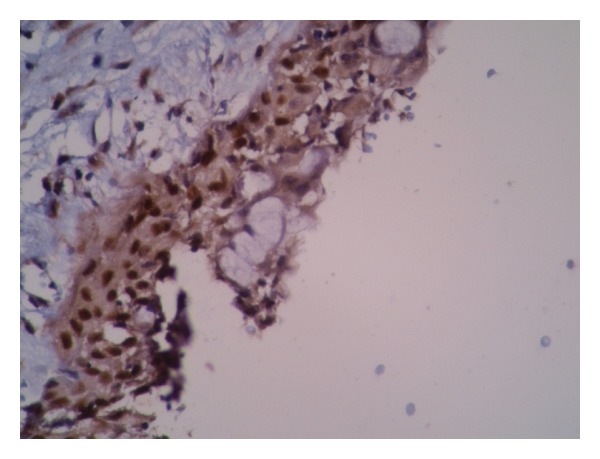
Dentigerous cyst with high expression of P63 in almost all epithelial layers. Mucous cells in the epithelial lining do not show any reaction (×200).

**Figure 3:**
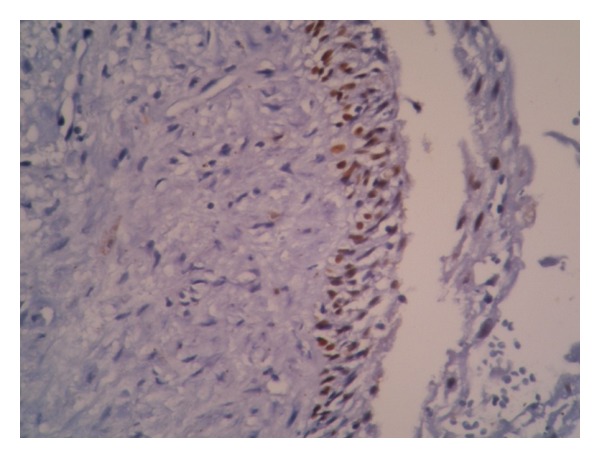
P63 expression in radicular cyst. Intense reactivity in basal and parabasal layers (×200).

**Figure 4:**
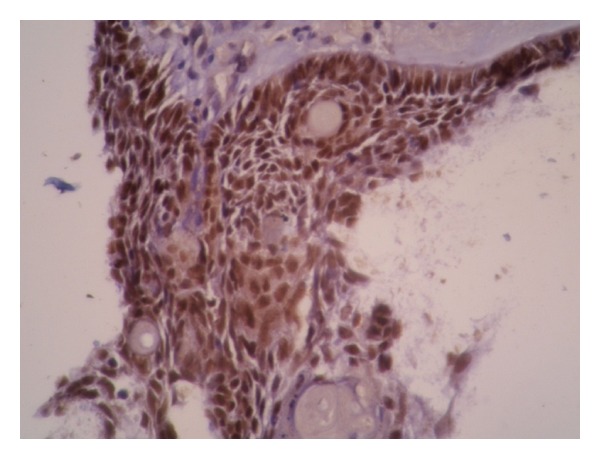
P63 expression in calcifying odontogenic cyst (×200). Ghost cells do not express this protein.

**Figure 5:**
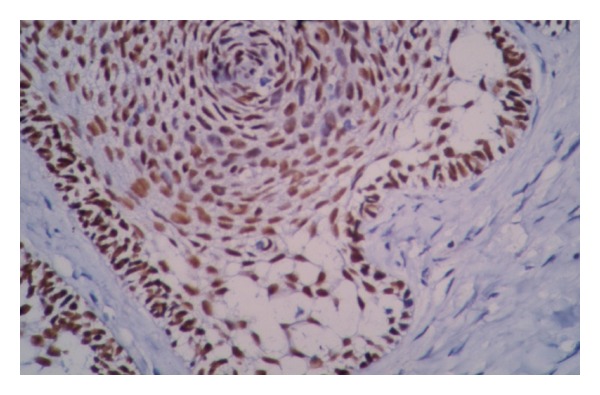
High expression of P63 in ameloblastoma. Peripheral columnar cells show more intense staining than stellate reticulum (×200).

P63 was expressed in all studied cases (*n* = 30). All lesions showed intense reactivity in odontogenic epithelium (Table 1). Immunostaining was found throughout the epithelial lining of OKC except the surface parakeratinized layer (Figure 1). In DCs, RCs mostly the basal and parabasal layers were positive for P63 (Figures 2, 3, and 4). Four cases of DCs also demonstrated intense reactivity in upper layers. No to weak reactivity was seen in the upper layers of RC. In addition, COC cases were immunostained in all layers of cyst. Ameloblastoma was intensely positive in peripheral cells. However, the reactivity in the stellate reticulum was weaker (Figure 5). With Kruskal-Wallis test no significant difference in the expression of P63 was observed between the lesions (*P* = 0.86). Mann-Whitney test revealed that there is significant difference between basal-parabasal and upper layers in odontogenic cysts (*P* < 0.001). Normal oral epithelium in sections also had positive immunostaining in basal-parabasal layers. The mucous cells of epithelial lining in DC and ghost cells of COC did not show any reaction to P63.

## 4. Discussion

P63, a member of P53 tumor suppressor gene family, plays a major role in the maintenance of epithelial stem cells and their terminal differentiation. P63 gene generates different protein isoforms (TA and ΔN) with different functions. ΔN P63 isoforms (lacking N-terminal transactivation domain) are involved in cell proliferation, while TAP63 isoforms (containing the transactivation domain) have a role in cell differentiation [2, 3]. In the absence of P63, stem cells and their progenies die by apoptosis, and the crippled stem cells are unable to bolster cell proliferation and self-renewal [7]. Upregulation of P63 has been also demonstrated in some malignancies [3]. In addition, P63 gene expression has been detected in the majority of tooth germ cells and dental epithelium throughout the bud and cap stages [1]. Investigators suggest that P63 is involved in epithelial differentiation during tooth development. Syndromes that are associated with P63 gene mutation have also various tooth abnormalities [3]. 

The results of this study is in accordance with other investigations of P63 expression in OKC which show positive immunoreactivity in all layers except for parakeratinized layer [4, 5, 7]. 

P63 reactivity was also evident in basal-parabasal layers of DC and RC. However, upper layers had weaker expression in these odontogenic cysts. Positivity of the upper layers in RC and DC has been reported to be 3–6% in other studies [5]. 

P63 expression in ameloblastoma was more intense in peripheral cells than in stellate reticulum. This finding is in accordance with Kumamoto et al. study [3] and may indicate the higher proliferative activity of peripheral cells in tumor islands of ameloblastoma.

Expression of P63 has been also investigated in other odontogenic lesions. Acanthomatous and granular cell types of ameloblastoma have lower P63 expression than common ameloblastoma [3]. In addition, adenomatoid odontogenic tumor has nuclear positivity for P63 indicating the basal characterization of tumor cells [8, 9]. Friedrich and Zustin described a case of Pindborg tumor with P63 expression in the vast majority of tumor cells [10]. In addition, Koutlas et al. reported three cases of Sclerosing odontogenic carcinoma with positive nuclear staining against P63 antibody [11]. Moreover, central granular cell odontogenic tumor demonstrates P63 expression in odontogenic epithelial islands. However, granular cells are negative [12]. This finding may indicate that this protein is not expressed in the mature differentiated cells. Gurgel et al. suggest that P63 expression reflects the immaturity of epithelial cells in keratinocytes of OKCs. They also stated that this protein participates in differentiation of epithelial cells [4]. In addition, we did not observe any positive reactivity in the mature keratinocytes of oral epithelium similar to previous studies [5, 13]. Therefore, it seems that P63 expression is limited to immature basal-parabasal layers of epithelium. 

Previous investigations show that all neoplastic and nonneoplastic odontogenic tissues express P63 protein [13]. Seyedmajidi et al. reported a higher expression of P63 in OKC than in DC and RC [14]. However, there was no significant difference between various odontogenic lesions in this study, which may be related to the different number of examined lesions in these two studies. Bello et al. also had an investigation of ameloblastoma and ameloblastic carcinoma. They showed that p63 expression is not significantly different between ameloblastoma and its malignant counterpart [15].

## 5. Conclusion

The expression of P63 in odontogenic cysts and tumors suggests that this protein is involved in differentiation and proliferation of odontogenic epithelial cells. It may also have a crucial role in tooth development. Moreover, P63 as a progenitor or basal cell marker is not expressed in mature cells. Also, it seems that this protein is not a useful marker in differentiation between odontogenic lesions with aggressive and nonaggressive behavior.

## Figures and Tables

**Table 1:** P63 expression in odontogenic lesions. No significant difference was observed between studied lesions (*P* = 0.86).

Pathologic lesion	*N*	Mean	±SD
OKC	9	98.33	2.5
AB	6	86.42	22.56
RAD	6	93.08	13.80
DC	6	93.75	10.46
COC	3	96.67	5.77
